# Short-Range Noncontact Sensors for Healthcare and Other Emerging Applications: A Review

**DOI:** 10.3390/s16081169

**Published:** 2016-07-26

**Authors:** Changzhan Gu

**Affiliations:** Google Inc., Mountain View, CA 94043, USA; changzhan@google.com or changzhan.gu@gmail.com

**Keywords:** radar, CW, FMCW, noncontact, biomedical, gesture, microwave, Doppler, vital sign

## Abstract

Short-range noncontact sensors are capable of remotely detecting the precise movements of the subjects or wirelessly estimating the distance from the sensor to the subject. They find wide applications in our day lives such as noncontact vital sign detection of heart beat and respiration, sleep monitoring, occupancy sensing, and gesture sensing. In recent years, short-range noncontact sensors are attracting more and more efforts from both academia and industry due to their vast applications. Compared to other radar architectures such as pulse radar and frequency-modulated continuous-wave (FMCW) radar, Doppler radar is gaining more popularity in terms of system integration and low-power operation. This paper reviews the recent technical advances in Doppler radars for healthcare applications, including system hardware improvement, digital signal processing, and chip integration. This paper also discusses the hybrid FMCW-interferometry radars and the emerging applications and the future trends.

## 1. Introduction

Since the first radars were developed as early as the 1930s [1], wireless sensing using radio frequency (RF) and microwave has become a well-established and mature field of engineering and applied sciences. The radar operation is based on the principle of electromagnetic backscattering [2]. The mainstream radar hardware and computational methods were developed for detection and tracking of large moving subjects at large distances, e.g. air-defense system and marine radars. In radar applications, “long range” refers to the distance between the radar and the target in the order of hundreds of meters or even kilometers, while “short range” usually means a few meters or less. It was in 1970s that the short-range radar technology was first introduced for healthcare applications of detecting human vital signs [3]. Without any sensor attached to the body, radar is able to wirelessly detect the tiny physiological movements due to heartbeat and respiration [4,5,6,7,8,9,10]. The non-contact detection of vital signs led to several potential applications such as sleep apnea detection [11], physiological monitoring [12], and earthquake rescue [13]. Owing to microwave penetration, the radar technology can detect human activities under debris after earthquakes [6,13]. In sleep apnea monitoring, if the subject’s vital sign signal disappears, an alarm is triggered to either wake up the subject or warn the subject’s guardians so they may take actions immediately [11]. Radar is a favorable approach for sleep monitoring, because it can accurately monitor the subject’s physiological signals and body movements without any devices being attached to the body. In contrast, the wearable technologies require the subject wear the device during sleep, which may affect the quality of sleeping [14]. Besides the healthcare applications, the radar sensor is also capable of detecting various mechanical movements in a noncontact manner [15,16,17], which leads to some emerging applications such as occupancy sensing [18,19] and gesture sensing for human-machine interaction [20,21]. 

According to the system architecture, the radar family is generally classified into the following categories: continuous-wave (CW) radar [22,23,24], frequency-modulated continuous-wave (FMCW) radar [25,26,27,28], and impulse radar [29,30,31]. Due to its broadband nature, impulse radar is not favorable in terms of system integration and low-power operation. It is not appealing in short-range applications because of the limited time delay from the short backscattering round trip. The short time delay requires an extremely high-speed switch and the broadband impulse needs to be captured by a power-hungry analog-to-digital converter (ADC) with very high speed [32]. In contrast, CW and FMCW allow higher level of system integration and lower power operation, which make them more attractive for mobile and portable applications [33,34,35]. FMCW is capable of both ranging and Doppler sensing but CW can only rely on Doppler information to track relative movements. In other words, FMCW radar can measure both absolute distance and relative displacement movements, while CW radar is only able to detect relative motions due to the lack of modulated spectral information [36,37,38]. Although both FMCW and CW radars can measure relative displacement motions, CW radar is superior to FMCW because of higher measurement accuracy, less complex hardware architecture and simpler signal processing approaches [39]. Therefore, for applications that only require relative displacement information, CW radar is a better option. Such scenarios include non-contact vital sign detection [40], sleep monitoring [41], mechanical vibration sensing [42], structural health monitoring [43], and so forth. On the other hand, FMCW radar is a better option for applications that need absolute distance information, such as monitoring the versatile life activities [44,45] and interactive gesture sensing [20,46,47].

The design of any radar system generally consists of two aspects: (1) radar hardware, including RF transceiver, baseband circuitry, processing unit, antenna, and so forth, and (2) digital signal processing (DSP) to parse the backscattered signal to recover the desired information. With growing interest of employing radar for short-range applications, many researchers have been pushing the technology boundaries in every aspect to try to make its applications ubiquitous in our daily lives. In the past two decades, various radar hardware architectures have been proposed, including direct-conversion quadrature radar receiver [48], digital intermediate-frequency (IF) receiver [24,49], injection locking radar [50,51], six-port radar [52], CW-FMCW hybrid radar [44,45], and millimeter wave radar on-chip [46,53]. Each of the architectures has its pros and cons and is suitable for certain specific applications. On the signal processing side, small angle approximation was first used to demodulate the phase-modulated motion information in CW radar [7,8]. Researchers later developed more advanced demodulation techniques including the complex demodulation [54] and the arctangent demodulation [55]. Other signal processing methods such as adaptive DC calibration [17,56], noise cancellation [57], distortion cancellation [58], and *I*/*Q* mismatch mitigation [59,60] have been proposed to make radar systems robust enough for practical applications. This paper reviews the recent technical advances in CW Doppler radar sensors for healthcare applications, including system hardware improvement, digital signal processing, and chip integration. Emerging applications and future trends will also be discussed. This paper also discusses the hybrid CW-FMCW radars and the emerging applications and future trends. 

This paper is organized as follows: Section 2 introduces the basic theory of single-tone CW Doppler radar, and the DSP techniques to extract the desired information; recent advancements in various aspects were reviewed and summarized in Section 3; Section 4 and Section 5 discuss the emerging applications and future trends respectively. Conclusions are presented in Section 6. 

## 2. Working Principle of CW Doppler Radar

Single-tone Doppler CW radar transmits an unmodulated RF signal to the target, which reflects part of the signal back to the radar receiver. The moving target modulates the carrier signal nonlinearly in the phase. Further DSP techniques can be used to recover the target’s movement from the nonlinear phase modulation. 

### 2.1. Theory

**Figure 1:**
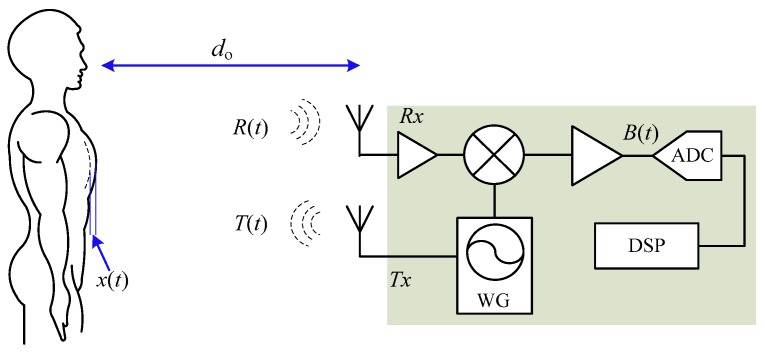
Simplified block diagram of CW/FMCW radar sensor and the mechanism of noncontact vital sign detection. WG: waveform generator.

The fundamental mechanism using single-tone CW radar for noncontact vital sign detection is shown in Figure 1. An unmodulated RF signal *T*(*t*) is generated by the waveform generator and transmitted via Tx antenna to the subject with a nominal distance of $d_{o}$ away:

(1) $$T\left(t\right)=A_{T}\cdot cos\left(2\pi ft+\theta \left(t\right)\right)$$

where *f* is the carrier frequency, $A_{T}$ is the power amplitude, and θ(t) is the phase noise from the waveform generator. The subject’s chest wall movement, which is induced by the respiration and heartbeat, modulates the RF signal and reflects part of the signal back to the radar. The signal received at radar Rx antenna is [8]:

(2) $$R\left(t\right)\approx A_{R}\cdot cos\left[2\pi ft-4\pi d_{o}/\lambda -4\pi x\left(t\right)/\lambda +\theta \left(t-2d_{o}/c\right)\right],$$

where $A_{R}$ is the received power, *λ* is the carrier wavelength, *c* is the speed of light in free space, x(t) is the chest wall movement including respiration and heartbeat, and $\theta \left(t-2d_{o}/c\right)$ is the phase noise with a delay of $2d_{o}/c$. 

As shown in Figure 1, the radar transceiver is a coherent structure that transmitter and receiver share the same signal source. That said, the same Tx signal is used as local oscillator (LO) signal to down-convert R(t) to baseband B(t):

(3) $$B\left(t\right)=cos\left[\theta_{o}+4\pi x\left(t\right)/\lambda +\Delta \theta \left(t\right)\right]$$

where $\theta_{o}=4\pi d_{o}/\lambda +\sigma$ is the summation of phase shift from the nominal distance $d_{o}$ and at the reflection surface, $\Delta \theta \left(t\right)=\theta \left(t\right)-\theta \left(t-2d_{o}/c\right)$ is the residual phase noise. 

Since both Tx and LO signals come from the same signal source, and they have the same phase noise characteristics [8]. In short range applications, the phase noise of the backscattered signal received by radar is strongly correlated with that of the LO signal. Therefore, phase noise from backscattering can be cancelled out by mixing with the LO signal so that the residual phase Δθ(t) in Equation (3) is usually negligible [34]. The range correlation effect makes it possible to cross over phase noise boundary to design high-sensitivity Doppler radar [8]. Due to the coherent nature and the fact that no spectral information is needed, a free running voltage controlled oscillator (VCO) is sufficient for Doppler radar. The waveform generator used in CW Doppler radar does not have to be circuitry based on strict frequency synthesis, such as phase-locked loop (PLL) or direct digital synthesizer (DDS), which dramatically reduces the complexity of the radar hardware system. 

The receiver structure shown in Figure 1 is direct-conversion homodyne. However, it is imperative to point out that other transceiver architectures are also being used in Doppler radar [24,49,50,51] and each of them has both advantages and disadvantages. For example, the zero-IF homodyne architecture allows high level of integration but it has strong dc offset at mixer output [61]; digital IF receiver makes it possible to completely eliminate *I*/*Q* mismatch at the cost of higher speed analog-to-digital converter (ADC) [24]. The selection of radar architecture is driven by specific applications and should be pondered over both hardware complexity and system performance specifications. Besides the radar architecture, the choice of carrier frequency is also of vital importance. People may infer from Equation (3) that the phase modulation is in inverse proportion to the carrier wavelength, so that the higher the frequency and better the detection sensitivity. However, researchers have found that, for CW Doppler radar, there is a strict relationship between carrier frequency and the displacement of the target movement [62]. Among all the carrier frequencies, 2.4 GHz and 5.8 GHz are probably the most popular ones likely due to the vast availability of low-cost RF/microwave components and integrated circuits (ICs) in the ISM bands. Studies have shown that both 2.4 GHz and 5.8 GHz Doppler radar are able to successfully detect human vital signs of respiration and heartbeat [33,34]. To optimize the detection of heartbeat that has small displacement of around one millimeter or even less, researchers have proposed higher frequency radars such as 24 GHz [62], millimeter-wave 60 GHz/96 GHz [53,63], and even 228 GHz [64]. Although higher frequencies are subject to higher path loss, they are beneficial in high modulation sensitivity and could the optimal choice for measuring small displacement in short range.

### 2.2. Demodulation Techniques

As can been seen from Equation (3), the movement information x(t) is nonlinearly modulated inside the cosine function. Special techniques are required to recover the actual movement information from nonlinear phase modulation.

- Small Angle Approximation

In vital sign detection, the displacement of respiration and heartbeat is very small compared to the carrier wavelength. If $\theta_{o}$ is an odd multiple of *π*/2, the radar baseband output, as shown in Equation (3), can be linearly approximated as:

(4) B(t)≈4πx(t)/λ+Δθ(t)

**Figure 2:**
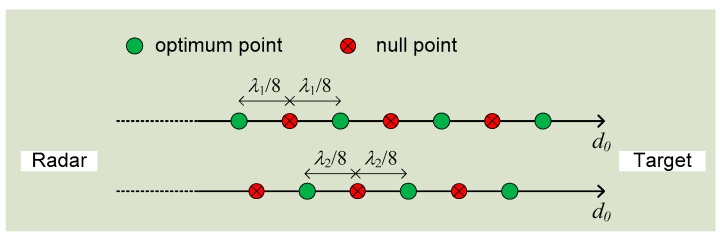
Frequency tuning using double sideband transmission to overcome the null point issue in Doppler radar detection. *λ*_1_ is is the wavelength of one sideband and *λ*_2_ is that of the other.

This is the optimum detection case since the baseband output is linearly proportional to the time-varying motion displacement x(t). However, as $\theta_{o}$ is a function of the distance to the target, there exist situations where $\theta_{o}$ happen to be an even multiple of *π*/2. In this case, the baseband output is no longer in proportion to x(t) and a “null point” occurs [65]. The “null point” issue not only means weak baseband output but also means inaccurate measurement results [55]. As the “null point” depends on $\theta_{o}$, which is determined by the target distance, the issue happens in an orderly fashion every *π*/4 from the radar. To overcome the null point issue, several effective approaches have been proposed. A frequency tuning technique was developed to adjust the “null points” distribution along the path away from radar [65]. It works by utilizing double sideband transmission in such a way that the null points from one sideband the optimum points from the other sideband can overlap each other, as shown below in Figure 2. 

**Figure 3:**
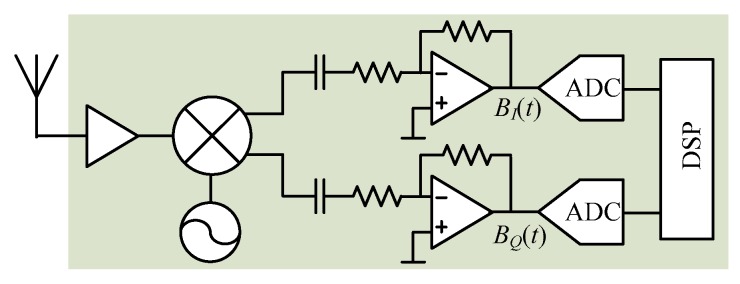
Quadrature Doppler radar receiver with *I*/*Q* channels.

The frequency tuning technique needs hardware tuning to achieve good detection accuracy, which is not very convenient. Another approach to solve the null point issue was proposed in [8] using a quadrature receiver. The quadrature *I*/*Q* channels ensure that there is always one channel that is not in the null detection point, without any need to tune the hardware. Unlike the one in Figure 1 which is a single channel receiver, Figure 3 shows the bock diagram of the quadrature receiver with *I*/*Q* channels.

Theoretically, the two *I*/*Q* channels at receiver baseband output are:

(5) $$B_{I}\left(t\right)=cos\left[\theta_{o}+4\pi x\left(t\right)/\lambda +\Delta \theta \left(t\right)\right]$$

(6) $$B_{Q}\left(t\right)=sin\left[\theta_{o}+4\pi x\left(t\right)/\lambda +\Delta \theta \left(t\right)\right]$$

When $\theta_{o}$ is an odd multiple of *π*/2, the *I* channel signal will be at the optimum point while the *Q* channel will be at the null point. Similarly, when $\theta_{o}\;$is an even multiple of *π*/2, the *I* channel will become a null point while the *Q* channel will be at the optimum point. Therefore, there is always one channel that is not at null point, ensuring good detection accuracy. In small angle approximation, the worst case is that $\theta_{o}$ is an integral multiple of *π*/4. In this case, none of the *I*/*Q* channels is at the optimum detection point.

- Arctangent demodulation

**Figure 4:**
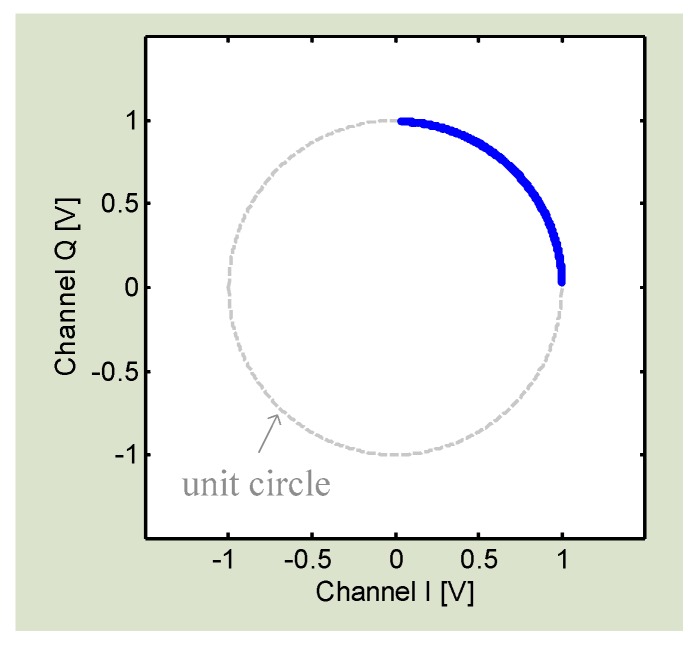
*I*/*Q* signals form an arch fitting with the unit circle in *I*/*Q* plane.

Small angle approximation is linear demodulation that only applies to small displacement. Arctangent demodulation is widely used as a nonlinear demodulation approach to precisely recover the full phase information from the nonlinear sinusoidal signal [55]. The actual *I*/*Q* signals are not the ideal sinusoidal signals as shown in Equations (5) and (6). In the practical radar system, the RF signal received at receiver front-end is amplified, filtered, converted directly to baseband *I*/*Q* signals, and then further boosted by the baseband amplifier. The *I*/*Q* signals before ADC are as follows [61]:

(7) $$B_{I}\left(t\right)=A_{I}\cdot cos\left[\theta_{o}+4\pi x\left(t\right)/\lambda +\Delta \theta \left(t\right)\right]+DC_{I}$$

(8) $$B_{Q}\left(t\right)=A_{Q}\cdot sin\left[\theta_{o}+4\pi x\left(t\right)/\lambda +\Delta \theta \left(t\right)\right]+DC_{Q}$$

where $A_{I}$ and $A_{Q}$ are the amplitude of *I*/*Q* channels, and $DC_{I}$ and $DC_{Q}$ are the DC offsets. The DC offset is from the reflections from the surrounding stationary objects and the circuit imperfections like mixer self-mixing [66]. It is seen that, if we can calibrate *I*/*Q* signals to remove the DC offsets $DC_{I}$ and $DC_{Q}$ and normalize the amplitude factors $A_{I}\;$and$\;A_{Q}$, the *I*/*Q* signals will become (5) and (6) which will fit with the unit circle in the *I*/*Q* plane as shown in Figure 4.

By using arctangent demodulation, the subject’s movement x(t) can be recovered:

(9) $$\theta_{o}+4\pi x\left(t\right)/\lambda +\Delta \theta \left(t\right)=arctan\left(\frac{B_{Q}\left(t\right)}{B_{I}\left(t\right)}\right)=arctan\left(\frac{sin\left[\theta_{o}+4\pi x\left(t\right)/\lambda +\Delta \theta \left(t\right)\right]}{cos\left[\theta_{o}+4\pi x\left(t\right)/\lambda +\Delta \theta \left(t\right)\right]}\right)$$

In a coherent Doppler CW radar, Δθ(t) is negligible due to range correlation effect [8]. $\theta_{o}$ is dependent on the fixed distance to the subject so it is a DC value which can be easily subtracted. Therefore, as long as the carrier wave length *λ* is known, x(t) can be easily recovered from Equation (9).

As discussed, DC calibration is required for effective arctangent demodulation. Since DC offset values are unpredictable, the calibration is challenging. The conventional method is by manually adjusting the $DC_{I}$/$DC_{Q}$ values as well as the amplitude factors $A_{I}$/$A_{Q}$ to fit the *I*/*Q* signals on the unit circle in the *I*/*Q* plane [67]. However, the manual circle fitting method is inefficient, sensitive to environmental changes, and sometimes inaccurate. Moreover, the estimation of calibration parameters is a subjective approach, which may vary from one person to another so that errors are inevitable. To have a robust DC calibration approach, researchers have proposed an algorithm based on compressed sensing using $\ell_{1}$ minimization [56]. Based on the input *I*/*Q* signals, the algorithm would automatically calculate the DC offset values $DC_{I}$/$DC_{Q}$ and a factor A to normalize the amplitudes. In this method, it assumes the there is no *I*/*Q* mismatch. The algorithm has been proved to be effective in arctangent demodulation and robust towards large interference [56].

**Figure 5:**
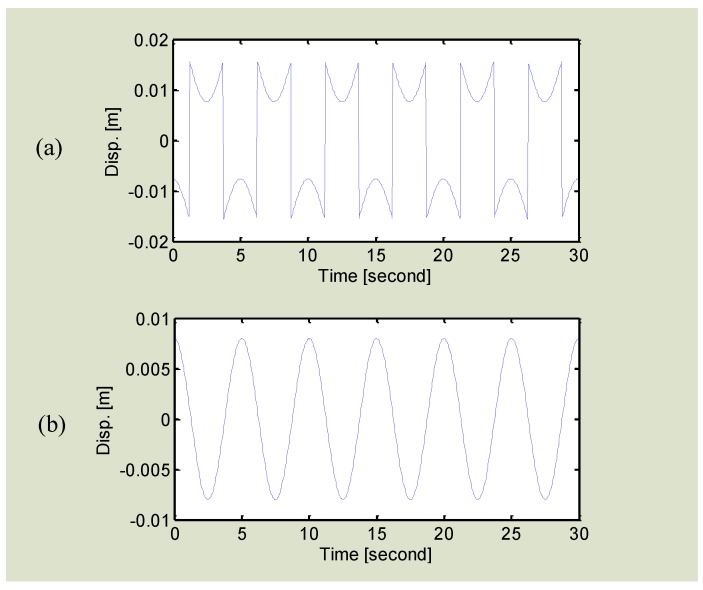
Demodulation of a 0.2 Hz 16 mm displacement movement in 2.4 GHz Doppler radar using (**a**) regular arctangent demodulation, and (**b**) extended DACM algorithm. Phase discontinuity is seen in (**a**) because the phase exceeds the range of (−*π*/2, +*π*/2).

Another challenge in arctangent demodulation is the phase discontinuity. Mathematically, an arctangent function only allows a native codomain range of (−*π*/2, +*π*/2). If the target motion displacement is relatively large compared to the carrier wavelength, the demodulation may exceed this range so that a phase discontinuity will occur. Figure 5a shows the demodulation of a 0.2 Hz 16 mm displacement movement in 2.4 GHz Doppler radar using the regular arctangent demodulation. It is seen that large discontinuity happens. Such discontinuity can be theoretically compensated in digital signal processing by shifting an integer multiple of *π*, which is a procedure called phase unwrapping [22]. However, in the real use case, it is actually not easy for a hardware or software to automatically make a judicious choice on which point needs a shift. It is extremely difficult when the vibration amplitude is large because it would cause severe discontinuity issues and the discontinuous data points may need to be compensated by different integer multiples of *π.* The other drawback of compensating by an integer multiple of *π* is the uncertainty of robustness to noise. To deal with the phase discontinuity problem, an extended differentiate and cross-multiply (DACM) algorithm is proposed in [68] for automatic phase unwrapping for phase reconstruction without ambiguities. In digital domain, the procedure of differentiation is usually approximately by forward difference:

(10) $$diff\left(\varphi \left[n\right]\right)=\frac{I\left[n\right]\cdot \frac{Q\left[n\right]-Q\left[n-1\right]}{\Delta t}-Q\left[n\right]\cdot \frac{I\left[n\right]-I\left[n-1\right]}{\Delta t}}{I{\left[n\right]}^{2}+Q{\left[n\right]}^{2}}\;$$

where φ[n], I[n], and Q[n] are digital representations as in φ(t)=arctan[Q(t)/I(t)], and Δt is sampling interval. With a further accumulation, the phase information φ[n] can be recovered as:

(11) $$\varphi \left[n\right]=\sum_{k=2}^{n}\frac{I\left[k\right]\cdot \left\{Q\left[k\right]-Q\left[k-1\right]\right\}-Q\left[k\right]\cdot \left\{I\left[k\right]-I\left[k-1\right]\right\}}{I{\left[k\right]}^{2}+Q{\left[k\right]}^{2}}$$

Instead of bothering with phase unwrapping, the DACM based phase demodulation technique allows the phase information to be directly retrieved from the calibrated *I*/*Q* signals without phase ambiguities. Figure 5b shows the processing of the same 0.2 Hz 16 mm displacement movement with the extended DACM algorithm. It is seen that the phase discontinuity problem is naturally solved. The DACM algorithm results in robust and accurate phase demodulation, regardless of the magnitude of vibration motions. It extends the linearity in nonlinear phase demodulation in Doppler radar sensing.

## 3. Recent Technical Advancements

The Doppler radar theory and the demodulation techniques laid the foundation for noncontact microwave sensing, based on which more advanced techniques have been developed to resolve various issues that hinder the ubiquitous applications of Doppler radar in our daily lives. 

### 3.1. Random Movement Cancellation

The first challenge that hinders the ubiquitous use of the radar technology may be the random body movement. The radar sensing is based on the illumination of radio frequency radiations to the subject. Since the antenna has certain radiation beam-width, all the targets within the radiation coverage would be illuminated and reflect signals back to the radar receiver. If the body is not stationary, its movement will also modulate the radar signal and will be mixed with heartbeat and respiration. The body movement is usually much larger than the heartbeat and respiration, which challenges the extraction of the expected vital sign signals [69,70]. The body movement may be removed in digital signal processing if it has a regular motion pattern. However, in practice, the body most likely moves randomly, which challenges the signal processing techniques to predict the precise motion trajectory. 

**Figure 6:**
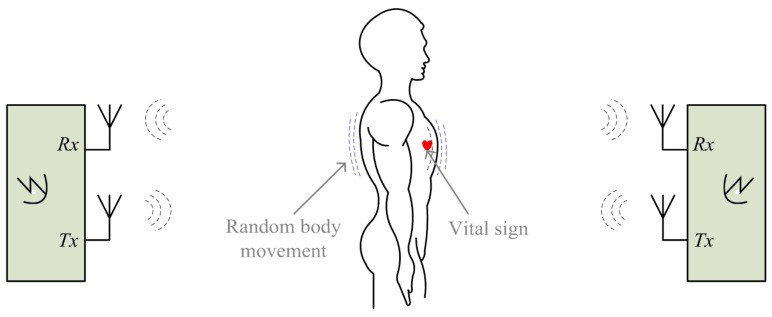
Setup of noncontact vital sign detection using two radars with random body movement cancellation.

Researchers have proposed several approaches to deal with the noise and artifacts such as the random body movement. A multi-radar system was introduced in [54] to detect the subject from different sides of the body. Figure 6 shows the setup of using two radars measuring from the back and the front of the body simultaneously. The two radars as shown in Figure 6 are identical and both use patch antennas with linear polarization. To mitigate the interference to each other, antennas on one of the radars can be rotated 90 degrees so that the two sets of antennas on the two radars are orthogonal [57]. As heartbeat and respiration are periodic movements with small amplitude relative to the radar wavelength, they have the same movement direction from the two radars’ perspective. However, for random body movement, when the body leans to one of the radars, it moves away from the other. That said, the distance change between the body and one radar is opposite as compared to that between the body and the other. By combining the measured signals from the two radars, the noise from random body movement can be mostly canceled [57]. A similar approach is proposed in [10] using two injection locking radars array for cancelling the body movement artifacts. By sweeping the radar’s carrier frequency or adjusting the subject’s position, it has experimentally proved that the vital sign signals can be successfully detected when the subject is jogging on a treadmill [10]. 

**Figure 7:**
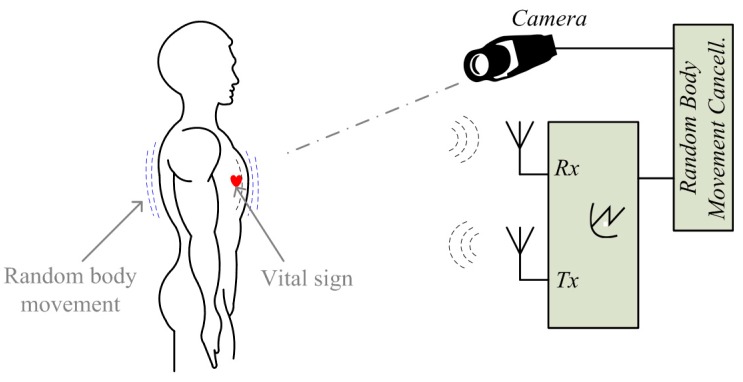
Setup of radar sensing with random body movement using a radar-camera hybrid system.

The sensors array approaches have apparent drawbacks. For example, they inevitably add to the system complexity, cost and power consumption. They are favorable for specific use cases where there is enough space to set up the sensors array. A radar-camera hybrid system using compensation was proposed in [69,70] to cancel the random body movement, the setup of which is shown in Figure 7. This approach proves the feasibility of using an ordinary smart phone camera to help to cancel the body movement. It is pretty attractive considering the nowadays’ popularity of smart phones. It works in such a way that the external phase information that is opposite to the phase information of random body movement is added in the radar received signals. Since the radar measured signal includes phase information of both vital sign and body movement, the added information could be used to only compensate the phase information of the body movement but retain the vital sign signals. Three motion compensation strategies were proposed in [70]: (a) phase compensation at RF front end using a phase shifter; (b) phase compensation using baseband complex signals; and (c) compensation after phase demodulation. Since the large body movement may produce large signals that may saturate the baseband circuitry, phase compensation using phase shifter at the radar RF front end helps to relieve the radar circuitry from potential saturation. The other two strategies are implemented in the baseband digital domain so they can be more precisely controlled to perform fine tuning to remove the artifacts. 

The aforementioned approaches are for compensating the body movement while the radar system is stationary. Researchers have also proposed approaches to tackle the movement of the radar platform itself, which can be useful as radar sensors are expected to be integrated in mobile platforms such as smart phone or tablet. For example, [71] demonstrated a complete compensated single transceiver radar system for vital sign detection in the presence of platform movement. By putting a custom designed tag near the subject, it produces a reference signal for the radar platform to compensate the movement of itself.

### 3.2. Signal Distortion Analysis and Compensation

**Figure 8:**
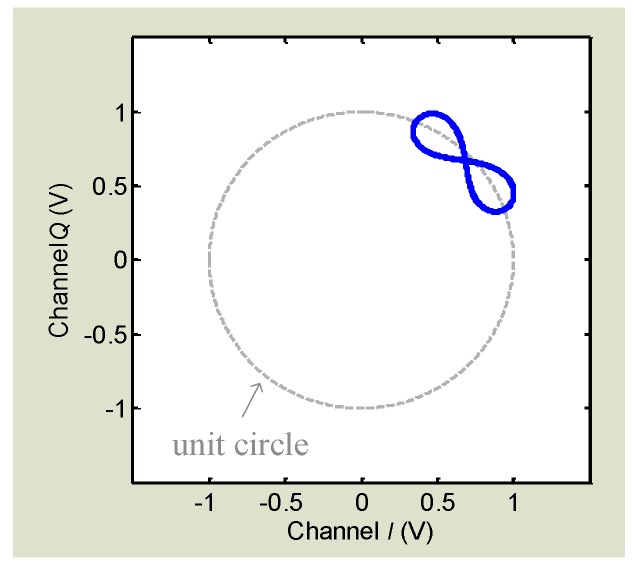
Signal distortion happens due to AC coupling. The trajectory is not an ideal arch but shows a ribbon-like shape in the *I*/*Q* plane.

Another challenge in Doppler radar sensing is the signal distortion while measuring subjects that are moving slowly. The signal pattern of the slow motions is often distorted. Homodyne receiver architecture with quadrature *I*/*Q* has been widely used for radar sensing, to avoid the null point problem and leverage the benefit of the range correlation effect [67]. However, due to circuit imperfections and clutter reflections from surrounding stationary objects, the homodyne receiver suffers from DC offset. AC coupling after mixer output is commonly used, probably the simplest way, to remove DC offset. The AC coupled baseband essentially has high pass characteristics, which results in signal distortion when the target motion has a very low frequency or a stationary moment [67]. For example, Figure 8 shows the distorted *I*/*Q* trajectory, which is due to AC coupling. 

As radar sensing is based on nonlinear phase modulation, the radar measured signal at baseband output can be expanded as Bessel function. For instance, a single tone sinusoidal movement x(t)=m·sin(ωt), where *m* is the amplitude and *ω* is the movement frequency, can be expressed as:

(12) $$B\left(t\right)=\overset{n=\infty }{\underset{n=-\infty }{\sum }}J_{n}\left(\frac{4\pi m}{\lambda }\right)\cdot cos\left(n\omega t+\varphi \right)\;$$

where φ is the total residual phase, *λ* is the wavelength, $J_{n}\left(x\right)\;$is the *n*th order Bessel function of the first kind. As can be seen in Equation (12), even for an ideal Doppler radar detecting a single-tone sinusoidal movement, harmonics will be created at the baseband output. If the target movement is slow or has stationary moment so that it falls into the stop-band of the active high-pass filter, the harmonics would be subject to different degrees of attenuation, owing to the slope characteristics of the high-pass filter. Therefore, signal distortion may happen due to the loss of the inherent spectrum characteristics, which shows as a ribbon-like shape in the *I*/*Q* plane. As discussed in the second section, DC calibration needs to be carried out before *I*/*Q* signals can be combined to perform arctangent demodulation to recover the motion information in the phase. However, the distorted ribbon trajectory challenges the calibration algorithm to find the accurate DC offset values as well as the center of the arch [67]. Moreover, the signal distortion may impact the demodulation accuracy because the length of the *I*/*Q* trajectory is strictly related to the magnitude of vibration [58]. 

**Figure 9:**
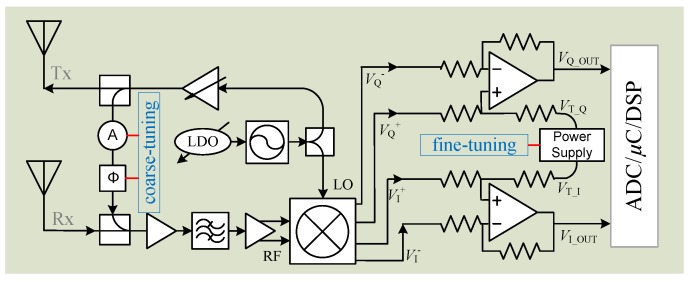
Block diagram of the Doppler radar with DC coupled architectures including RF coarse-tuning and baseband fine-tuning.

To eliminate the signal distortion, a straightforward method is to avoid using the AC coupled baseband structure and employ the DC coupled architecture for radar hardware [61,66]. The DC coupled baseband is all-pass architecture, not like high-pass in AC coupling, so that all the harmonics from nonlinear phase modulation will undergo the same degree of attenuation or amplification, which ensures no signal distortion. To employ DC coupling, hardware modifications need to be made in order that the DC offset at mixer output will not saturate the following stages. Figure 9 shows the block diagram of the DC coupled Doppler radar system, as proposed in [61]. The radar system was designed with adaptive DC tuning stages including RF coarse-tuning and baseband fine-tuning. As we know, a major part of the DC offset is from direct coupling from the transmitter to receiver resulting in the mixer self-mixing. To deal with that, the RF tuning was implemented using a path of an attenuator and a phase shifter at the RF front end of the Doppler radar system, as shown in Figure 8. By doing so, it adds a portion of the transmitter signal back to the receiver so as to cancel out most of the DC offset at mixer output. To further calibrate the remaining DC offset and to minimize the quantization noise impact from ADC, the baseband fine-tuning circuitry is added to adaptively adjust the amplifier bias to the desired level. With the two tuning stages, it allows both high gain amplification and maximum dynamic range the baseband stage. With the proposed DC tuning architectures, the Doppler radar is able to precisely measure the low-frequency movement even if the motion trajectory has stationary moment. 

**Figure 10:**
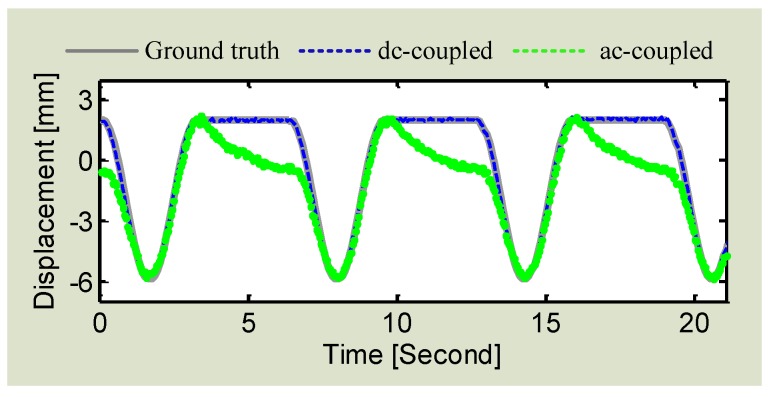
Experimental results of using both DC radar and AC radar to measure the actuator motion which is sinusoidal with a stationary moment in between two cycles.

The proposed DC coupled radar was experimentally verified in the lab environment. Both the DC radar and AC radar were used to measure the actuator that was programmed to perform sinusoidal movement with a short period of stationary moment in between two adjacent cycles. The experimental results, as shown in Figure 10, illustrates that the DC radar matches with the actuator very well by successfully preserving the stationary information. However, owing to the fact that the AC coupling capacitors cannot hold the charge for a long time and they tend to discharge over the stationary moment, the stationary information is distorted in AC radar. 

**Figure 11:**
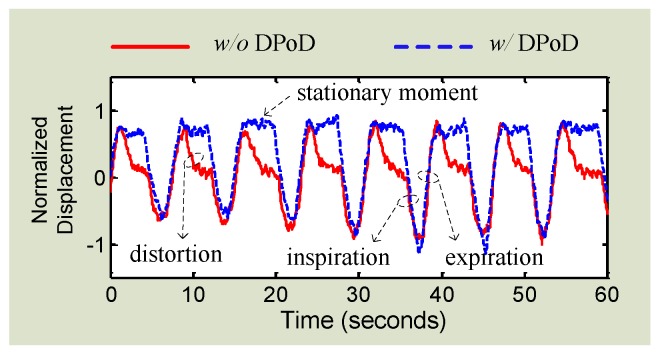
Respiration motion measured by AC coupled Doppler radar with and without using the proposed DPoD technique.

Using DC coupled radar inevitably adds to the hardware complexity. Several approaches have been proposed to use AC coupled radar for accurate displacement measurement without adding to radar architecture complexity. Reference [72] presents a method of calibrating *I*/*Q* signals in AC coupled radar to achieve high accuracy displacement measurement. The calibration is based on data-based imbalance compensation [59] and radius correction [73]. Nevertheless, the limitation is that a severely distorted ribbon-shaped *I*/*Q* trajectory may not be corrected to recover the actual displacement due to the fact that the displacement is directly translated from the length of the trajectory [58]. A more advanced technique of digital post-processing (DPoD) was proposed to compensate for the signal distortions in the digital baseband domain [58]. Without any cumbersome hardware modification, the simple quadrature direct-conversion receiver architecture can be used to detect the complete pattern of slow periodic motions. As many authors may know, pre-distortion technique is widely used in power amplifier’s linearization. In contrast to that, the proposed digital DPoD technique applies signal compensation in the digital baseband to recover the signal information that may be lost in an AC coupled receiver. The signal distortion is compensated in the digital domain by an algorithm whose system response is the inverse function of that of the high-pass filter at AC coupled baseband. That being said, the DPoD technique tends to “linearize” the high-pass characteristics of the AC coupling so that the AC coupled baseband imparts virtual “all-pass” characteristics to the input signals. Therefore, the signal integrity can be preserved using the low-cost low-complexity AC coupled architecture without any hardware modifications. The DPoD technique was experimentally evaluated by using an AC coupled radar to measure a healthy adult at rest. It is known that the respiratory motion for an adult at rest includes a short period of stationary moment, which means that the respiration tends to rest for a moment at the end of expiration [33]. The experimental result in Figure 11 shows that, without DPoD, the information of stationary moment is lost so the respiration signal was distorted. After applying DPoD, the stationary moment is recovered so the complete respiratory patter is restored. 

### 3.3. I/Q Mismatch Mitigation

Besides distortion in the conventional AC coupled receiver baseband, another source of distortion comes from the *I*/*Q* imbalance in quadrature radar receiver [59,60]. The *I*/*Q* imbalance includes amplitude imbalance and phase imbalance, which are studied in details in [74]. With the advancement of integrated circuit (IC) technologies, many of nowadays’ RF components have good *I*/*Q* balance performance. For example, the quadrature mixer used in [58], i.e., Skyworks73009, has a maximum *I*/*Q* amplitude imbalance of 0.3 dB and a typical phase error of only 1°. However, lower quality radars at reasonable price are still popular for some applications, such as the automotive anti-collision system and the automotive doors. For instance, the radar module used in [60] has amplitude imbalance of 1.25° and phase imbalance of 23°. Therefore, it is worthwhile exploring techniques to mitigate *I*/*Q* imbalance in inexpensive radar modules for high-accuracy displacement measurement.

The Gram-Schmidt (GS) or ellipse correction method is commonly used in coherent optical communication systems to compensate the *I*/*Q* imbalance [59,60]. This method uses a least square fitting algorithm to match the *I*/*Q* data to an ellipse, based on which the orthogonality of the *I*/*Q* signals are corrected by a known transformation [59]. A method using GS to estimate the amplitude and phase imbalances in Doppler radar was proposed in [59]. However, this method requires modifying the radar hardware by adding an external voltage controlled phase shifter between antenna and radar transceiver. Moreover, it requires more than one full circle of data in the *I*/*Q* plane, while most cases of radar displacement sensing only occupy a portion of the unit circle. A data-based method called “ellipse fitting” that requires no hardware modifications has been proposed in [59]. With a mechanical target moving linearly in front of the radar, this method estimates the imbalance parameters from the ellipse that the data forms in the *I*/*Q* plane. Since all the data points along the arc in *I*/*Q* plane contribute to the estimation, this method is more robust. To fit the ellipse parameters to the data, there are generally two methods: algebraic fitting or geometric fitting, the latter of which is known to be more accurate and robust. The method in [60] is based on algebraic ellipse-fitting. In [60], the performances of algebraic and geometric fitting methods are compared in different conditions in radar sensing and the *I*/*Q* imbalance estimation problem is studied in details. The Levenberg-Marquardt (LM) method is also proposed in [60] as an easy imbalance estimation approach to address the *I*/*Q* imbalance problem in radar sensing. 

### 3.4. Hardware System Integration

In addition to the technical advancements on the system side and the signal processing side, researchers have also made tremendous efforts to develop advanced techniques for integrating radar sensor systems on miniature printed circuit boards or on silicon as a chip substrate. Doppler radar systems built up using instruments [24] or off-the-shelf connectorized RF modules [55] are usually bulky in size and hungry in power consumption, making them inconvenient for portable applications. Research efforts have been made to integrate the radar system on board and on chip. Owing to the advancements of the IC technologies in this century, there are numerous commercially available RF chips in the market, especially in the market of mobile platforms such as smart phones and tablets. In healthcare applications, the vital sign signals of respiration and heartbeat are usually very weak vibration motions with amplitudes of a few millimeters or even less. Unlike the common observation that higher frequency lead to better performance because of deeper phase modulation, it has been demonstrated that radar modulation sensitivity is not proportional to the carrier frequency but is strongly correlated with the ratio between the vibration amplitude and the carrier frequency [62]. It is expected that frequency in the 20 GHz range may result in the optimal heartbeat detection for most people [62]. However, the board level system integration needs to leverage not only the sensing performance but also the chip availability, cost, and form factor. Since various wireless technologies are using the ISM bands of 2.4 GHz and 5 GHz, such as Bluetooth and WiFi, there are ample RF ICs available in the market at reasonably low price. Integrating radar systems at lower frequencies is more cost effective and can meet the performance requirement as long as the system is carefully designed. 

**Figure 12:**
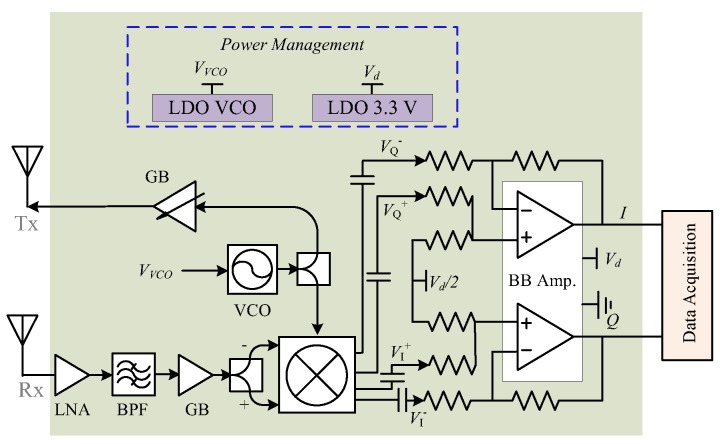
Block diagram of the 2.4 GHz Doppler radar integrated on the printed circuit board (as shown in the shaded area).

**Figure 13:**
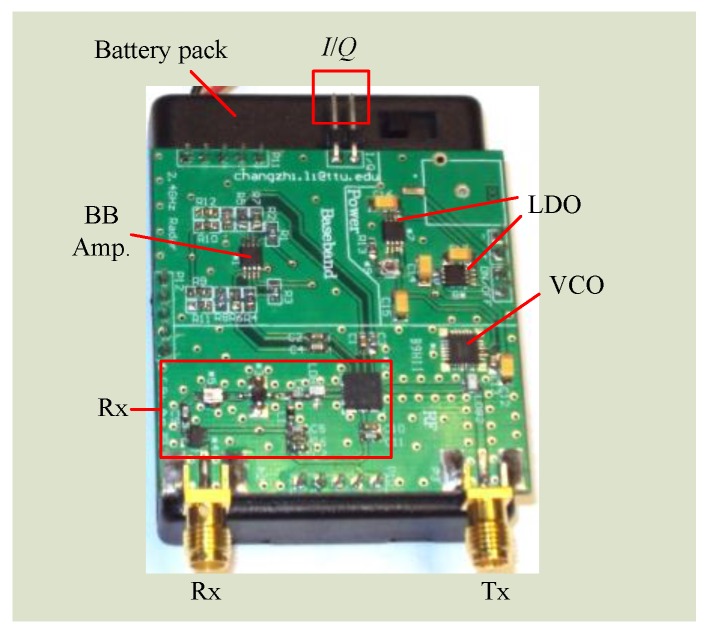
Picture of the 2.4 GHz Doppler radar sensor.

Although WiFi 802.11n/ac employs 5 GHz, there are a lot more lower-cost RF ICs in the 2.4 GHz band. From a cost perspective, it is a better approach to make the system work at 2.4 GHz. Figure 12 shows the block diagram of a miniature 2.4 GHz radar system designed by researchers at Texas Tech University (Lubbock, TX, USA). The system-on-board has a form factor of 5 cm by 5 cm. Figure 13 is the picture of the fabricated and assembled 2.4 GHz Doppler radar. The radar transceiver was designed as a homodyne architecture with AC coupled baseband. It is a coherent system because both transmitter and receiver share the same free-running voltage controlled oscillator (VCO). The distance between the subject and the radar is usually at most several meters in healthcare applications, which is important for the range correlation effect to stand. Due to range correlation, phase noise from VCO can be cancelled out at receiver so that it does not affect the low-frequency respiration and heartbeat signals. The VCO is free-running so it transmits a single tone signal. The VCO output is divided equally via a surface-mount balun into two parts. One part of the signal is amplified and directly sent out via Tx antenna to the subject. In life applications, such a Tx amplifier is optional depending on the desired detection range. If an Tx amplifier is employed, special attentions should be put on the direct coupling of the strong Tx signal back to Rx front end, which may saturate the Rx chain. The other part of VCO output is fed into the quadrature mixer as the local oscillation (LO) signal to convert the received RF signal down to baseband. 

The Doppler radar receiver mainly consists of a low noise amplifier (LNA), a band-pass filter, a gain block, and a quadrature mixer. The weak vital sign signal requires that LNA have good noise figure and decent gain to suppress the noise contributions from the following stages. To block out of band interferers, a commercial 2.4 GHz band pass filter with bandwidth of 100 MHz is used. The 2.4 GHz ISM band is full of many other wireless technologies, such as WiFi, Bluetooth, microwave oven, etc. It is possible that the 2.4 GHz interferences may fall in the radar band. However, the co-existence is usually not a severe issue as long as the radar sensor keeps a reasonable distance away from the noise source. As the vital sign signal is very narrow bandwidth, e.g. respiration is less than half a Hz and heartbeat is just over a Hz, the baseband amplifier is configured to be very narrow bandwidth to let the vital sign signals pass but will block the interferers in the 2.4 GHz ISM band. The baseband amplifier essentially has narrow bandwidth with a gain-bandwidth-product of less than a few MHz. If the gain is configured to be high such as 200, the baseband bandwidth would be minimized to just pass vital sign signals. The 2.4 GHz ISM technologies like WiFi have duty cycle in transmission, which further helps to avoid the co-existence issue. A quadrature mixer is used to directly convert the RF signal to baseband. Moreover, the mixer’s differential output helps to better reject the common-mode noise and even-order harmonic distortions. The power management ICs includes a 3.3 V 500 mA low-dropout regulator (LDO), which supplies power to all RF and baseband ICs, and a tunable LDO, which is used to tune the VCO to be within the desired frequency band. The *I*/*Q* signals from baseband amplifier’s output would be sampled by the data acquisition system and further processed off-line. 

**Figure 14:**
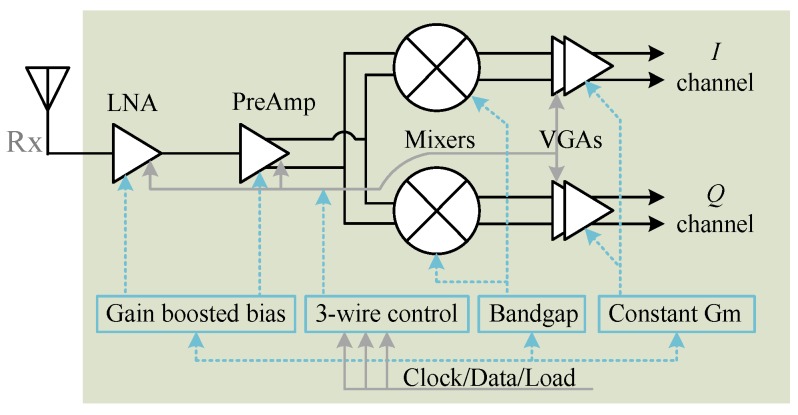
Simplified block diagram of the 5 GHz software-configurable Doppler radar sensor.

**Figure 15:**
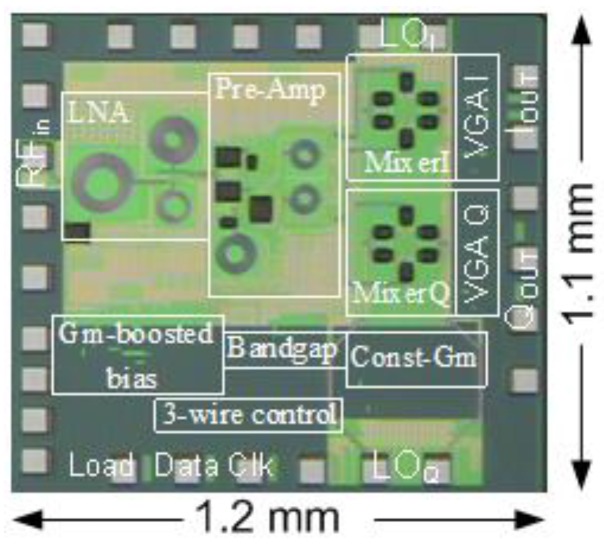
Microphotograph of the 5 GHz software-configurable Doppler radar sensor [75].

Besides the board level system integration, researchers have also made efforts to integrate the radar system on silicon [8,26,35,46,48]. The first effort of chip design for a biomedical radar dates back to the early 2000s [48]. The vital sign signal is very weak so the Doppler radar needs to be highly sensitive. To make the radar technology closer to real life applications, a 5 GHz software-configurable high-sensitivity Doppler radar system-on-chip (SoC) was proposed in [75]. Figure 14 and Figure 15 illustrate the block diagram and the microphotograph of the high-sensitivity radar chip, respectively. A 3-wire control bus makes the radar software-configurable to set the operation point and the detection range for optimal performance. The proposed radar SoC is highly integrated that all the RF and analog functions are integrated on-chip. The baseband *I*/*Q* output can be directly sampled for digital signal processing. To achieve high sensitivity, special considerations have been taken on important issues such as flicker noise, baseband bandwidth, and the link budget. Analog-digital co-design was also carried out to optimize the system performance. The link budge analysis, which is dedicated to physiological sensing, shows that large gains in the receiver chain and low flicker noise in the down-conversion mixer and baseband are of key importance for the high sensitivity performance in detecting slow signals of respiration and heartbeat. The experimental results show that the designed radar sensor chip has a high sensitivity of better than −101 dBm for sensing physiological signals when there is no random body movement.

### 3.5. Hybrid FMCW-Interferometry Radar

Although multiple critical advancements have been made for the conventional microwave radar sensor, it still has some limitations to fully monitor the real-time life activities. The biggest challenge is how to provide the concurrent information of distance and displacement at low cost. The main stream radar architectures, that is, Doppler (interferometry) radars, impulse radars, FMCW radars, and stepped frequency-modulated continuous-wave (SFCW) radars, cannot meet the requirements. For example, Doppler radars uses single-tone RF signal to obtain phase history. Although they have been widely used for high-precision displacement measurement, they can hardly detect the absolute distance information. There is a challenge for a single Doppler radar to distinguish multiple targets in light-of-sight view of the radar. The impulse, FMCW and SFCW radars are capable of detecting the absolute distance. Nevertheless, large bandwidth is needed to achieve the range resolution needed for monitoring in-home life activities and many of them are forced to work at high frequencies such as millimeter wave to archive the required large bandwidth. The associated problems such as linearity, cost, complexity and high attenuation prevent them from ubiquitous applications. 

**Figure 16:**
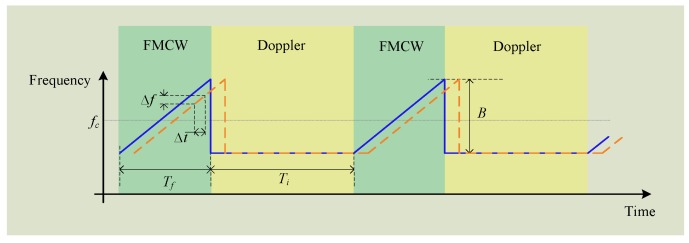
Transmitted signal of the FMCW-interferometry radar.

A hybrid FMCW-interferometry radar was proposed in [44,45] for precise 2-D positioning and life activities surveillance. The proposed hybrid radar works in the 5.8 GHz ISM band with a 160 MHz bandwidth. Based on CW architecture, the FMCW mode and the interferometry mode are incorporated together. The FMCW mode is responsible for detecting absolute range while the interferometry mode is sued for sensing the relative displacement motions such as the tiny physiological signals. The two hybrid modes can share the same RF front end architecture as both of them are based on CW operation. To achieve good Tx and Rx isolation, two separate antennas are used for Tx and Rx, respectively. A turntable allows the antennas to scan over 360° to get the 2-D information. Figure 16 shows the proposed transmitted signal in time domain. The FMCW signal has the same amplitude with the Doppler signal in time domain. The transmitted signal is basically a sequence of chirp period embedded into single-tone interferometry signal. The interferotry signal has a fixed operating frequency, while the FMCW signal is a chirp signal that is an up-ramp linear frequency-modulated signal. At radar receiver, by mixing a copy of the transmitted signal with that backscattered from the subject, the baseband output contains a series of the FMCW signal and the interferometry signal. Strict clocking synchronization is necessary within the radar system between the signal generator, the digitizer, and the baseband signal processer. If the clocking is not well synchronized, the difference between two clocks will accumulate and eventually misalign the actual location of the beat signals that are used for extracting the range information. The range information is calculated based on the baseband beat signal. 

**Figure 17:**
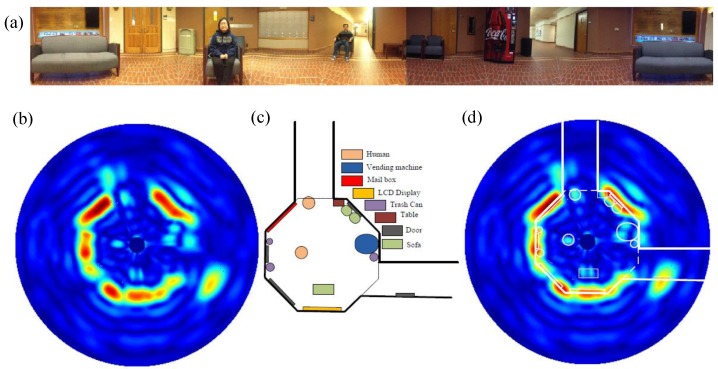
(**a**) The panorama photograph of the experimental environment; (**b**) 2-D location map obtained by FMCW detection mode; (**c**) room layout and objects distribution in the test room; (**d**) the comparison between detection and the actual location.

As shown in Figure 16, the beat signal occur with a fixed interval, so that once the location of one beat signal is determined, those of the following beat signals can be easily predicted based on the FMCW burst interval. Details about the signal processing approaches to extract the range and displacement information can be found in [45]. Figure 17 shows the real 2-D location and life activities monitoring. Figure 17a is a panorama photograph of the experimental room, which has an octagon shape and is filled with several objects such as sofa, a table, a vending machine, and a trash can. Figure 17b shows the 2-D location map obtained by FMCW detection mode and the comparison between detection and the actual location is shown in Figure 17c. The outline of the room is clearly plot on the map as well as two people sitting at the directions of 11 o’clock and 9 o’clock. The sofas and refrigerator are dim in the map, because the strength of the reflected single depends on both the objects’ radar cross section and the material. The sofa is made of soft cloth which electromagnetic waves can penetrate through so it makes a weak reflector. The smooth cover on the refrigerator serves as a mirror that tilts backscattering signal to other directions. By comparing the 2-D map with the actual location, the excellent match illustrates that the radar has good accuracy in indicating the position of the objects in real life environment. 

### 3.6. Concurrent Detection of Range and Displacement

The hybrid FMCW-interferometry radar is capable to detect the absolute range and the relative displacement at the same time. The conventional Doppler radar is only able to capture the Doppler signature due to the lack of bandwidth information. However, by modifying the hardware system architecture, the conventional Doppler radar could be equipped with the capability of ranging. A Doppler radar system with adaptive beam-steering antenna was presented in [76]. With single-tone unmodualted transmission, the proposed radar is capable of concurrent detection of vibration and distance at the same time. The detection theory is based on the fact that the DC offset in the baseband signal contains useful information on distance. In prior works [77], dynamic DC offset tracking has been realized to adaptively calibrate out the DC offset for motion imaging and large displacement sensing. The radar system proposed in [76] extracts the phase from the DC offsets and processes the phase difference between propagation paths at different antenna beam-steering angles. The absolute distance information could be accurately calculated with a fast beam-switching antenna in homodyne architecture [76]. 

As shown in Equation (3), the residual phase in radar baseband signal is given by:

(13) $$\phi_{1}=4\pi d_{o}/\lambda +\varepsilon_{o}+\Delta \theta \left(t\right)$$

**Figure 18:**
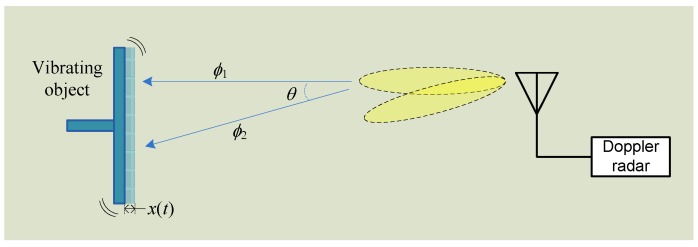
The Doppler radar with beam-steering antenna could radiate the signal to the target in two directions with an angle of *θ*.

Since the phase noise of the radar system can be cancelled out due to the range correlation effect [8], the value of the term Δθ(t) is ≪1 and can usually be neglected. $\varepsilon_{o}$ is the summation of the constant phase shift from the reflection surface and the constant phase delay in Rx. Therefore, the total phase residue is a function of the distance $d_{o}$. The distance information could be extracted by using an adaptive beam-steering antenna as shown in Figure 18. 

The Doppler radar with beam-steering antenna is able to radiate the beams in two directions alternatively and fast. If the angle of two radiation beams is *θ*, the total residual phase with the angle of *θ* of the beam would be:

(14) $$\phi_{2}=\frac{4\pi d_{o}\cdot sec\left(\theta \right)}{\lambda }+\varepsilon_{o}+\gamma +\Delta \theta {\left(t\right)}^{'}$$

where γ is the phase difference of the shifter on the antenna when the beam steers. $\Delta \theta {\left(t\right)}^{'}$ can be negligible due to phase correlation effect. The difference between Equations (13) and (14) is:

(15) $$\phi_{2}-\phi_{1}=\frac{4\pi d_{o}}{\lambda }\left(\sec\left(\theta \right)-1\right)+\varepsilon_{o}+\gamma$$

Once the phase difference as in Equation (15) is known, the distance can be determined as:

(16) $$d_{o}=\left[\left(\phi_{2}-\phi_{1}\right)-\left(\varepsilon_{o}+\gamma \right)\right]\cdot \frac{\lambda }{4\pi \left(\sec\left(\theta \right)-1\right)}$$

**Figure 19:**
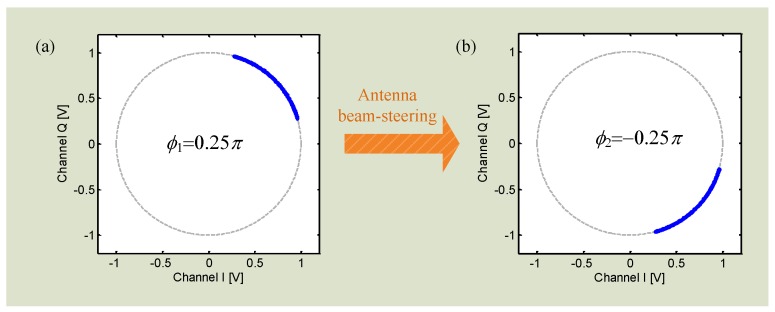
The location of the *I*/*Q* trajectory changes as the distance between radar and target changes. The distance change is realized by antenna beam-steering.

The total residual phase can be calculated with the desired DC offset values. The phase difference can then be obtained. Figure 19 illustrates the simulation result of the locations of the *I*/*Q* trajectory when the antenna steers beams. The initial total residual phase is 0.25*π* and the trajectory sits in the first quadrant. However, when the antenna beam is steered, the trajectory moves to the forth quadrant and the total residual phase is now −0.25*π*. The phase difference of 0.5*π* could then be used to predict the absolute distance according to Equation (16). 

One specific application of the technique is for structural health monitoring to wireless detect the infrastructural deformations due to man-made vibrations or natural seismic events, which might not be noticeable through visual inspection. 

## 4. Emerging Applications

### 4.1. Guesture Sensing

In addition to the various healthcare applications using Doppler radar [78,79], the radar-related technologies find applications in gesture sensing for human-machine interaction. The interaction between human and smart phones is mainly based on push-button or touch screen technologies, which are reliable and widely perceived as the standard features of the smart phones. However, as smart phones and other smart devices such as tablets are being increasingly used in our daily lives, we are seeing limitations in the interaction interface. For example, we may want to change the music or pick up a phone when we are in the kitchen busy with cooking or cleaning dishes, or we need to change the radio channel or tune up volume while we are driving. In all these scenarios, pushing the touch screen is difficult and can be distracting. 

As the advancement of technologies, camera-based video technology has emerged as a new gesture sensing approach that serves as a great supplementation to the traditional human-computer interaction methods. However, camera-based gesture sensing has several inherent drawbacks. First, gesture sensing using camera is based on video processing of the recorded digital frames, which usually requires powerful process unit. It is therefore not power efficient. Moreover, many mobile platforms does not have powerful processing unit and they are often powered by battery pack, which does not allow them to use camera for gesture sensing. Second, some of the existing camera approaches require special types of camera. For instance, the camera system used in Microsoft’s Kinect sensor is an array of cameras that capture the depth map of the action target. It is not only expensive but also bulky. Third, the optical camera is sensitive to the ambient light. Gesture sensing won’t work in weak light or darkness. Forth, optical signal cannot penetrate obstacles such as pocket cloth between the phone and the band [80]. 

As wireless power transfer (WPT) and near field communication (NFC) using inductive coils are standardized and have become competitive features for smart phones in recent years, it also opens up new opportunities to use inductive coupling to develop new human-machine interface [21]. Either WPT or NFC is based on the electromagnetic coupling between the planar coils. In WPT or NFC, there are coils in both the base station and the mobile device. The coil in the base station acts as the transmitter antenna that transfer power to the receive coil antenna in the mobile device via inductive coupling. In interactive sensing, instead of using the coils for power transfer or conveying message, the coil sends alternating magnetic field to the human hand and the hand motion alternates the field to interact with the mobile device. The hand movement in the magnetic field of the coil in front of the mobile device changes the coil’s conductivity distribution, which lead to the change of the effective coil impedance. The impedance change will result in a drift in the resonant frequency, which can be measured using an LC coupled oscillator structure. Thus, the WPT or NFC coil can detect hand movements, which makes it possible for use to interact with the smart device without touching them. 

Besides the sensing hardware, machine learning and gesture recognition algorithms are also important for successful interactive sensing. As gesture differs from one person to another, machine learning can be employed as an important approach to improve the accuracy and response time in gesture recognition. Based on the user’s specific hand movements, parameters in digital signal processing and hardware configuration could be adjusted specifically so as to quickly predict the incoming gesture command. Classifiers are necessary to distinguish different action gestures, such as swiping the band from left to right or from right to left. Advanced machine learning and classifiers need to be developed to recognize micro gestures such as click and virtual slider.

A recent advancement in interactive sensing is Google’s Project Soli, which is a new interactive sensing technology for gesture recognition based on millimeter wave radar [47,80]. The Soli sensor was designed as a 60 GHz FMCW radar and the entire system including antennas was integrated as a compact solid-state semiconductor device, i.e. a radar sensor chip. The radar chip is a miniature and low-power device that has no moving parts and can be manufactured inexpensively at scale. The Soli sensor has been demonstrated to be an indeed viable, powerful and promising technology that can enhance and improve user interaction. A broad range of applications of this technology can be envisioned, including wearable and mobile interaction, gaming control, smart home electronics, internet of things, augmented reality (AR) and virtual reality (VR), and other exciting applications. Soli envisions a future of a truly ubiquitous gesture interaction language that would allow users to control all smart computing devices with one universal gesture set, without bothering touching. Soli is still in early stage of development. However, once the technology is mastered, it is expected that the physical interface would disappear and our hand will naturally become the only interfacing device we would ever need for gesture sensing to control all things. 

### 4.2. Occupancy Sensing

Occupancy sensing is employed not only for security purposes, but also for the control of lighting, heating, ventilation, air conditioning (HVAC), and other presence-related loads and demands in commercial and residential spaces [19]. It is expected that, by occupancy sensing, the conservation of energy can be realized through the automated shutdown of loads no long needed when a space is unoccupied. In recent years, energy efficiency and energy conservation are becoming more and more important attracting increasingly attention. A nearly 50% increase is projected to happen in global energy use by 2025 [19]. Occupancy sensors can save up to 80% of energy used for lighting and HVAC systems, according to the research [19], which will also result in huge financial savings. 

The common types of occupancy sensors include the passive infrared and the ultrasonic sensors. However, both of them are subject to high rates of false alarms and failure to detect stationary subjects. These two types of sensors are effectively motion sensors but not the best sensors for detecting human presence. In the past two decades, researchers in the radar sensor field has made tremendous technological progress to make the sensor low-cost, highly integrated while has high accuracy. All the technical advancements have made the Doppler radar technology feasible for occupancy sensing. Moreover, the integration of processing unit and RF transceiver on one single chip, which is often called system-on-chip (SoC), provides a new platform for combination of sensing, processing, and communication. The high level system integration lays the foundation for a smart sensor network in the future smart home and could be the key part of the sensing system of the internet of things (IoT). Besides detecting the large motions of human activities, Doppler radar sensor is also capable of detecting the weak vital sign signals, which provides a promising approach to overcome the problems of false alarms and dead spots in the conventional sensors. Radar sensors make true presence detection possible. Doppler radar can discern the physiological motion from non-human periodic motion that could otherwise trigger false positives. It can prevent false negatives by detecting heart beat and respiration rates in the case of stationary human subjects, while the existing other occupancy sensors lack accuracy to do so. 

## 5. Future Trends

Doppler radar has undergone tremendous advancements in the past two decades in nearly all of the technical aspects, including transceiver architecture, antenna optimization, system minimization, chip integration, demodulation techniques, and digital signal processing. Due to the vast potential applications of the technology, we can expect to see the advent of more advances in the years to come. For biomedical applications, random body movement is an issue that prevents Doppler radar from ubiquitous applications. Although some approaches have been proposed to tackle the issue, as discussed in previous sections, more technical advances are needed to solve the body movement issue in some specific use cases such as in-home monitoring. From the system hardware perspective, it is expected to have higher level of chip integration including RF, baseband and the processing unit. As the antenna takes much space, designing a sensor chip at high frequency such as in the millimeter wave range could be an option to minimize the antenna size and even integrate the antenna together on chip or in package. Normally, a Doppler radar can only provide Doppler signature which is the relative motion information, but does not have ranging capability to detect the absolute distance. Other radar techniques, including impulse and FMCW, are good at ranging but not superior at measuring relative motions. Therefore, it is expected to design hybrid radar system that combines the merits of each radar technology. Also, chip integration of the hybrid radar sensor is expected so that the sensor chip could be put into mobile platforms such as smart phone or watch. 

## 6. Conclusions

Owing to its capability of precisely detecting small amplitude vibration motions, Doppler radar technology has been used for noncontact vital sign detection. In the past few decades, especially in the new century, Doppler radar has attracted significant attention from researchers across the world and numerous important advancements have been made to push the technology further into practical applications. Various hardware architectures have been proposed to tackle different scenarios and issues associated with biomedical applications. Although some radar systems are still in the early prototype stage, they have proven the possibility and laid the foundation for the following development to make the technology ubiquitous in life applications. The random body movement was studied and several approaches such as the camera-radar hybrid system were proposed to solve the issue. The digital post-distortion technique was proposed to solve the signal distortion problem that happens in AC coupled radar, without need to modify the hardware architecture. To overcome the Doppler radar incapability of ranging, a hybrid FMCW-interferometry radar was designed to be able to measure distance and displacement at the same time. While radar hardware is important, technical advancements have also happened on the signal processing side. For example, the techniques to mitigate *I*/*Q* mismatch make the radar sensor more robust in displacement sensing. The radar technique is also promising in numerous emerging applications such as gesture sensing for human-machine interaction and occupancy sensing. It is expected that, with the joint efforts from researchers in the radar community, more advancements will be seen in Doppler radar to push the technology into more practical applications. Some emerging applications such as gesture sensing seem futuristic now but could become reality soon. 

## References

[1] Watson-Watt R. (1945). Radar in War and in Peace. Nature.

[2] Skolnik M.I. (1962). Introduction to radar. Radar Handbook 2.

[3] Lin J.C. (1975). Non-invasive microwave measurement of respiration. IEEE Proc..

[4] Gu C., Li C. (2015). Assessment of Human Respiration Patterns via Noncontact Sensing Using Doppler Multi-Radar System. Sensors.

[5] Li C., Chen F., Jin J., Lv H., Li S., Lu G., Wang J. (2015). A Method for Remotely Sensing Vital Signs of Human Subjects Outdoors. Sensors.

[6] Chen K.-M., Misra D., Wang H., Chuang H.-R., Postow E. (1986). An X-band microwave life-detection system. IEEE Trans. Biomed. Eng..

[7] Massagram W., Lubecke V., Høst-Madsen A., Boric-Lubecke O. (2009). Assessment of Heart Rate Variability and Respiratory Sinus Arrhythmia via Doppler Radar. IEEE Trans. Microw. Theory Tech..

[8] Droitcour A.D., Boric-Lubecke O., Lubecke V.M., Lin J., Kovac G.T. (2004). Range correlation and I/Q performance benefits in single-chip silicon Doppler radars for noncontact cardiopulmonary monitoring. IEEE Trans. Microw. Theory Tech..

[9] Li C., Xiao Y., Lin J. (2006). Experiment and spectral analysis of a low-power -band heartbeat detector measuring from four sides of a human body. IEEE Trans. Microw. Theory Tech..

[10] Wang F.-K., Horng T.-S., Peng K.-C., Jau J.-K., Li J.-Y., Chen C.-C. (2011). Single-antenna Doppler radars using self and mutual injection locking for vital sign detection with random body movement cancellation. IEEE Trans. Microw. Theory Tech..

[11] Li C., Lin J., Xiao Y. Robust overnight monitoring of human vital signs by a non-contact respiration and heartbeat detector. *Engineering in Medicine and Biology Society*, Proceedings of the 28th Annual International Conference of the IEEE.

[12] Lin J.C. (1992). Microwave sensing of physiological movement and volume change: A review. Bioelectromagnetics.

[13] Chen K.-M., Huang Y., Zhang J., Norman A. (2000). Microwave life-detection systems for searching human subjects under earthquake rubble or behind barrier. IEEE Trans. Biomed. Eng..

[14] Yilmaz T., Foster R., Hao Y. (2010). Detecting Vital Signs with Wearable Wireless Sensors. Sensors.

[15] Kim S., Nguyen C. (2003). A displacement measurement technique using millimeter-wave interferometry. IEEE Trans. Microw. Theory Tech..

[16] Wang T., Zheng N., Xin J., Ma Z. (2011). Integrating Millimeter Wave Radar with a Monocular Vision Sensor for On-Road Obstacle Detection Applications. Sensors.

[17] Guan S., Rice J., Li C., Gu C. (2014). Advanced DC Offset Calibration Strategy for Structural Health Monitoring Based on Portable CW Radar Sensor. IEEE Trans. Instrum. Meas..

[18] Yavari E., Jou H., Lubecke V., Boric-Lubecke O. Doppler radar sensor for occupancy monitoring. *Biomedical Wireless Technologies, Networks, and Sensing Systems (BioWireleSS)*, Proceedings of the 2013 IEEE Topical Conference.

[19] Yavari E., Song C., Lubecke V., Boric-Lubecke O. (2014). Is There Anybody in There?: Intelligent Radar Occupancy Sensors. IEEE Microw. Mag..

[20] Wang F.-K., Tang M.-C., Chiu Y.-C., Horng T.-S. (2015). Gesture Sensing Using Retransmitted Wireless Communication Signals Based on Doppler Radar Technology. IEEE Trans. Microw. Theory Tech..

[21] Liu C., Gu C., Li C. Non-contact Hand Interaction with Smart Phones Using the Wireless Power Transfer Features. Proceedings of the IEEE Radio and Wireless Symposium (RWS).

[22] Kim S., Nguyen C. (2004). On the development of a multifunction millimeter-wave sensor for displacement sensing and low-velocity measurement. IEEE Trans. Microw. Theory Tech..

[23] Droitcour A., Lubecke V., Lin J., Boric-Lubecke O. A microwave radio for Doppler radar sensing of vital signs. *Microwave Symposium Digest*, Proceedings of the 2001 IEEE MTT-S International.

[24] Gu C., Li C., Lin J., Long J., Huangfu J., Ran L. (2010). Instrument-based noncontact Doppler radar vital sign detection system using heterodyne digital quadrature demodulation architecture. IEEE Trans. Instrum. Meas..

[25] Vivet D., Checchin P., Chapuis R. (2013). Localization and Mapping Using Only a Rotating FMCW Radar Sensor. Sensors.

[26] Tessmann A., Kudszus S., Feltgen T., Riessle M., Sklarczyk C., Haydl W.-H. (2002). Compact single-chip W-band FMCW radar modules for commercial high-resolution sensor applications. IEEE Trans. Microw. Theory Tech..

[27] Li Y.-A., Hung M.-H., Huang S.-J., Lee J. A fully integrated 77 GHz FMCW radar system in 65nm CMOS. Proceedings of the 2010 IEEE International Solid-State Circuits Conference—(ISSCC).

[28] Li Z., Wu K. (2008). 24-GHz frequency-modulation continuous-wave radar front-end system-on-substrate. IEEE Trans. Microw. Theory Tech..

[29] Zhang C., Kuhn M.-J., Merkl B.-C., Fathy A.-E., Mahfouz M.-R. (2010). Real-time noncoherent UWB positioning radar with millimeter range accuracy: Theory and experiment. IEEE Trans. Microw. Theory Tech..

[30] Zhang X., Xi X., Li M., Wu D. (2016). Comparison of Impulse Radar and Spread-Spectrum Radar in Through-Wall Imaging. IEEE Trans. Microw. Theo. Tech..

[31] Chudobiak W.J., Gray R., Wight J.S. (1978). A nanosecond impulse X-band radar. IEEE Proc..

[32] Wang Y., Yang Y., Fathy A.E. Reconfigurable ultra-wide band see-through-wall imaging radar system. Proceedings of the 2009 IEEE Antennas and Propagation Society International Symposium.

[33] Gu C., Li C. (2014). From Tumor Targeting to Speech Monitoring: Accurate Respiratory Monitoring Using Medical Continuous-Wave Radar Sensors. IEEE Microw. Mag..

[34] Li C., Lubecke V., Boric-Lubecke O., Lin J. (2013). A Review on Recent Advances in Doppler Radar Sensors for Noncontact Healthcare Monitoring. IEEE Trans. Microw. Theory Tech..

[35] Li C., Xiao Y., Lin J. (2008). A 5 GHz double-sideband radar sensor chip in 0.18 m CMOS for non-contact vital sign detection. IEEE Microw. Wire. Compon. Lett..

[36] Yan Y., Li C., Rice J.A., Lin J. Wavelength division sensing RF vibrometer. *Microwave Symposium Digest (MTT)*, Proceedings of the 2011 IEEE/MTT-S International Microwave Symposium.

[37] Anghel A., Vasile G., Cacoveanu R., Ioana C., Ciochina S. (2014). Short-Range Wideband FMCW Radar for Millimetric Displacement Measurements. IEEE Trans. Geosci. Remote Sens..

[38] Li Y., O’Young S. (2015). Method of doubling range resolution without increasing bandwidth in FMCW radar. Electron. Lett..

[39] Lu L., Li C., Rice J.A. A software-defined multifunctional radar sensor for linear and reciprocal displacement measurement. *Wireless Sensors and Sensor Networks (WiSNet)*, Proceedings of the 2011 IEEE Topical Conference.

[40] Gu C., Li R., Li C., Jiang S.-B. Doppler radar respiration measurement for gated lung cancer radiotherapy. *Biomedical Wireless Technologies, Networks, and Sensing Systems (BioWireleSS)*, Proceedings of the 2011 IEEE Topical Conference.

[41] Huang M.-C., Liu J., Xu W., Gu C., Li C., Sarrafzadeh M. (2016). A Self-Calibrating Radar Sensor System Design for Measuring Vital Signs. IEEE Trans. Biomed. Circuits Syst..

[42] Yan Y., Cattafesta L., Li C., Lin J. (2011). Analysis of Detection Methods and Realization of a Real-time Monitoring RF Vibrometer. IEEE Trans. Microw. Theory Tech..

[43] Gu C., Wang G., Rice J.A., Li C. Interferometric Radar Sensor with Active Transponders for Signal Boosting and Clutter Rejection in Structural Health Monitoring. *Microwave Symposium Digest (MTT)*, Proceedings of the 2012 IEEE/MTT-S International Microwave Symposium.

[44] Wang G., Gu C., Inoue T., Li C. Hybrid FMCW-interferometry radar system in the 5.8 GHz ISM band for indoor precise position and motion detection. *Microwave Symposium Digest (IMS)*, Proceedings of the 2013 IEEE MTT-S International Microwave Symposium.

[45] Wang G., Gu C., Inoue T., Li C. (2014). A Hybrid FMCW-Interferometry Radar for Indoor Precise Positioning and Versatile Life Activity Monitoring. IEEE Trans. Microw. Theory Tech..

[46] Nasr I., Karagozler E., Poupyrev I., Trotta S. A Highly Integrated 60-GHz 6-Channel Transceiver Chip in 0.35 μm SiGe Technology for Smart Sensing and Short-Range Communications. Proceedings of the 2015 IEEE Compound Semiconductor Integrated Circuit Symposium (CSICS).

[47] Google’s Project Soli. https://atap.google.com/soli/.

[48] Droitcour A.-D., Boric-Lubecke O., Lubecke V.-M., Lin J. 0.25/spl mu/m CMOS and BiCMOS single-chip direct-conversion Doppler radar for remote sensing of vital signs. Solid-State Circuits Conference, 2002. Digest of Technical Papers. ISSCC. In Proceedings of the 2002 IEEE International.

[49] Yavari E., Boric-Lubecke O. Low IF demodulation for physiological pulse Doppler radar. Proceedings of the 2014 IEEE MTT-S International Microwave Symposium.

[50] Wang F.-K., Chou Y.-R., Chiu Y.-C., Horng T.-S. (2015). Chest-Worn Health Monitor Based on a Bistatic Self-Injection-Locked Radar. IEEE Trans. Microwave Theory Tech..

[51] Horng T.-S. Self-injection-locked radar: An advance in continuous-wave technology for emerging radar systems. Proceedings of the 2013 Asia-Pacific Microwave Conference Proceedings.

[52] Gabor V., Lindner S., Barbon F., Mann S., Hofmann M., Duda A., Weigel R., Koelpin A. (2013). Six-port radar sensor for remote respiration rate and heartbeat vital-sign monitoring. IEEE Trans. Microwa. Theory Tech..

[53] Kao T., Yan Y., Shen T., Chen A., Lin J. (2013). Design and Analysis of a 60-GHz CMOS Doppler Micro-Radar System-in-Package for Vital-Sign and Vibration Detection. IEEE Trans. Microw. Theory Tech..

[54] Li C., Lin J. Complex signal demodulation and random body movement cancellation techniques for non-contact vital sign detection. *Microwave Symposium Digest*, Proceedings of the 2008 IEEE MTT-S International Microwave Symposium.

[55] Park B.K., Boric-Lubecke O., Lubecke V.M. (2007). Arctangent demodulation with DC offset compensation in quadrature Doppler radar receiver systems. IEEE Trans. Microw. Theory Tech..

[56] Xu W., Gu C., Li C., Sarrafzadeh M. (2012). Robust Doppler radar demodulation via compressed sensing. IET Electron. Lett..

[57] Li C., Lin J. (2008). Random body movement cancellation in Doppler radar vital sign detection. IEEE Trans. Microw. Theory Tech..

[58] Gu C., Peng Z., Li C. (2016). High-Precision Motion Detection Using Low-Complexity Doppler Radar with Digital Post-Distortion Technique. IEEE Trans. Microw. Theory Tech..

[59] Singh A., Gao X., Yavari E., Zakrzewski M., Cao X.H., Lubecke V., Boric-Lubecke O. (2013). Data-based quadrature imbalance compensationfor a CW Doppler radar system. IEEE Trans. Microw. Theory Tech..

[60] Zakrzewski M., Singh A., Yavari E., Gao X., Boric-Lubecke O., Vanhala J., Palovuori K. (2013). Quadrature Imbalance Compensation With Ellipse-Fitting Methods for Microwave Radar Physiological Sensing. IEEE Trans. Microw. Theory Tech..

[61] Gu C., Li R., Zhang H., Fung A., Torres C., Jiang S., Li C. (2012). Accurate respiration measurement using DC-coupled continuous-wave radar sensor for motion-adaptive cancer radiotherapy. IEEE Trans. Biomed. Eng..

[62] Gu C., Inoue T., Li C. (2013). Analysis and Experiment on the Modulation Sensitivity of Doppler Radar Vibration Measurement. IEEE Microw. Wirel. Compon. Lett..

[63] Bakhtiari S., Elmer T.W., Cox N.M., Gopalsami N., Raptis A.C., Liao S., Mikhelson I., Sahakian A.V. (2012). Compact millimeter-wave sensor for remote monitoring of vital signs. IEEE Trans. Instrum. Meas..

[64] Gatesman A.J., Danylov A., Goyette T.M., Dickinson J.C., Giles R.H., Goodhue W., Waldman J., Nixon W.E., Hoen W. (2006). Terahertz behavior of optical components and common materials—Art. no. 62120E. Terahertz Military Security Appl. IV.

[65] Xiao Y., Lin J., Boric-Lubecke O., Lubecke M. (2006). Frequency-tuning technique for remote detection of heartbeat and respiration using low-power double-sideband transmission in the *K*a-band. IEEE Trans. Microw. Theory Tech..

[66] Gu C., Li C. (2012). DC coupled CW radar sensor using fine-tuning adaptive feedback loop. IET Electron. Lett..

[67] Gu C., Li C. (2012). Frequency-Selective Distortion in Continuous-Wave Radar Displacement Sensor. IET Electron. Lett..

[68] Wang J., Wang X., Chen L., Huangfu J., Li C., Ran L. (2014). Non-contact Distance and Amplitude Independent Vibration Measurement Based on an Extended DACM Algorithm. IEEE Trans. Instrum. Meas..

[69] Gu C., Wang G., Inoue T., Li C. Doppler radar vital sign detection with random body movement cancellation based on adaptive phase compensation. *Microwave Symposium Digest (IMS)*, Proceedings of the 2013 IEEE MTT-S International Microwave Symposium.

[70] Gu C., Wang G., Inoue T., Li C. (2013). A Hybrid Radar-Camera Sensing System with Phase Compensation for Random Body Movement Cancellation in Doppler Vital Sign Detection. IEEE Trans. Microw. Theory Tech..

[71] Rahman A., Yavari E., Singh A., Lubecke V.-M., Lubecke O.-B. (2015). A Low-IF Tag-Based Motion Compensation Technique for Mobile Doppler Radar Life Signs Monitoring. IEEE Trans. Microw. Theory Tech..

[72] Gao X., Boric-Lubecke O. AC Coupled Quadrature Doppler Radar Displacement Estimation. *Microwave Symposium Digest (IMS)*, Proceedings of the 2015 IEEE MTT-S International Microwave Symposium.

[73] Gao X., Singh A., Yavari E., Lubecke V., Boric-Lubecke O. Non-contact Displacement Estimation Using Doppler Radar. Proceedings of the 2012 34th Annual International Conference of the IEEE Engineering in Medicine and Biology Society.

[74] Yan Y., Li C., Lin J. Effects of I/Q mismatch on measurement of periodic movement using a Doppler radar sensor. Proceedings of the 2010 IEEE Radio and Wireless Symposium (RWS).

[75] Li C., Yu X., Lee C.-M., Li D., Ran L., Lin J. (2010). High-sensitivity software-configurable 5.8-GHz radar sensor receiver chip in 0.13-m CMOS for noncontact vital sign detection. IEEE Trans. Microw. Theory Tech..

[76] Nieh C.-M., Wei C., Lin J. (2015). Concurrent Detection of Vibration and Distance Using Unmodulated CW Doppler Vibration Radar with an Adaptive Beam-Steering Antenna. IEEE Trans. Microw. Theory Tech..

[77] Gu C., Xu W., Wang G., Inoue T., Rice J., Ran L., Li C. (2014). Noncontact Large-Scale Displacement Tracking: Doppler Radar for Water Level Gauging. IEEE Microw. Wire. Componen. Lett..

[78] Li C., Lin J. (2013). Microwave Noncontact Motion Sensing and Analysis.

[79] Boric-Lubecke O., Lubecke V.M., Droitcour A.D., Park B.K., Singh A. (2015). Doppler Radar Physiological Sensing.

[80] Lien J., Gillian N., Karagozler M.E., Amihood P., Schwesig C., Olson E., Raja H., Poupyrev I. (2016). Soli: Ubiquitous Gesture Sensing with Millimeter Wave Radar. ACM Trans. Graph..

